# Supplementary material for: Are carbon emissions trading and green financial instruments synergistic? -Comprehensive quantitative research based on content analysis
Source: PLoS One. 2024 Mar 7;19(3):e0298601. doi: 10.1371/journal.pone.0298601 (PMC10919601; doi:10.1371/journal.pone.0298601)
Supplement: S1 Data — (ZIP) [file pone.0298601.s001.zip › Data/地级市Z_F工作报告.zip!/║╙▒▒/║Γ╦«╩╨/└┤╫╘╣1⁄2╓┌║┼í╛┬φ┐╦╩2╛▌═°í┐_2018.docx]

各位代表：

现在，我代表市人民政府，向大会报告工作，请予审议，并请市政协委员和列席会议的同志提出意见。

一、2017年工作回顾

过去的一年，是新一届政府的开局之年，也是衡水市坚持以习近平新时代中国特色社会主义思想为指导、加快经济强市美丽衡水建设进程中很不平凡的一年。面对艰巨繁重的改革发展稳定任务，在省委、省政府和市委的坚强领导下，在市人大、市政协及社会各界的监督支持下，我们以迎接学习宣传贯彻党的十九大为动力，以新发展理念统领全局，紧紧围绕“一三五七”总体部署，全力稳增长、促改革、调结构、治污染、惠民生、防风险，经济社会发展呈现稳中有进的良好态势。

综合实力显著增强。全年实现地区生产总值1550.1亿元，较上年增长7.2%。规模以上工业增加值495.2亿元，增长5%。固定资产投资1300.3亿元，增长5.6%。社会消费品零售总额751.5亿元，增长11.3%。全部财政收入191.4亿元，其中一般公共预算收入103.5亿元，分别增长10.5%和8.1%。城镇居民人均可支配收入26195元，农村居民人均可支配收入11194元，分别增长10.1%和11.2%。多数指标增速高于全省平均水平，综合经济实力迈上新的台阶。

结构调整步伐加快。确立“4+4”产业体系，出台支持企业技术创新、振兴工业实体经济等系列文件，一业一策推动传统特色产业转型升级。全市新增规模以上工业企业120家，高新技术企业70家。装备制造业和高新技术产业增加值分别增长15.5%、8.7%，占规模以上工业的比重分别达到31.7%、19.6%。瑞丰动力在香港主板上市，养元饮品上市已经证监会核准，老白干成功并购联想旗下丰联酒业。深入实施创新驱动发展战略，依托衡水科技谷、蓝火基地等科技转化平台，推进与中科院、中国农科院等科研机构深度合作，全市新增工程技术研究中心7家，衡水高新区入列京南国家科技成果转移转化示范区和京津冀现代制造业平台。积极发展以“文旅农”融合为方向的旅游业和以电子商务、快递物流为主的生产性服务业，旅游总收入、接待游客增幅均为全省第一，桃城、冀州、深州、武强成功入选首批省级全域旅游示范区创建单位，服务业对经济增长的贡献率达到66%。突出“三农”工作夯实发展根基，粮食生产稳定在35亿公斤以上，畜牧、蔬菜、果品三大产业稳步提升，注册家庭农场、登记农民专业合作社和农村土地流转率均居全省首位，畜禽养殖废弃物资源化利用的“安平模式”受到国务院领导充分肯定。过去一年，我们坚持以供给侧结构性改革为主线，狠抓经济转型升级，新产业新业态新模式蓬勃发展，新旧动能转换顺畅接续，经济发展的质量、效益得到进一步提升。

重点项目成果丰硕。深入开展重点项目突破年活动，狠抓有质量的投资，大上优质高效项目。新开工亿元以上项目291项，超出年度任务的38.6%。140项省市重点项目提前两个月完成全年投资计划，超出年度任务的39.4%。正大肉鸡全产业链项目等开工建设，总投资116亿元的北京和平铝业铝型材深加工等大项目相继谈定落地，新谋划10亿元以上项目176项。项目规模、层次、质量持续突破，充分发挥了有效投资对经济发展“压舱石”的作用，为跨越赶超积蓄了后劲。

城乡建设统筹推进。围绕“四个一百”目标，推进主城区组团式发展，加快补齐短板、完善功能。大力实施“3个10”市政重点工程，火车站广场综合改造、文化艺术中心、大广高速衡水湖连接线等竣工投用，植物园、奥体中心等开工建设，综合管廊建设顺利推进。“双违”整治攻坚行动提前完成省定目标。集中解决房地产开发遗留问题，促进房地产市场健康发展。智慧城市建设步入快车道。以“六个一”工程和“两馆一中心”为重点，谋划县城建设项目356个，完成投资191.2亿元。省级园林县城实现全覆盖，枣强、深州、阜城入围全省“洁净城市”。新开工“三区同建”项目14个，“三区同建”示范点达到65个，在全省率先实现农村新型社区省级示范点县域全覆盖。城镇建设全面展开，城乡面貌焕然一新。

综合改革蹄疾步稳。国家级中小城市综合改革试点工作顺利完成，得到国家发改委高度评价。农业农村综合改革继续领跑全省，冀州颁发全省第一本新版农村土地承包经营权证，农业水价综合改革不断深化，饶阳建立农村集体经济发展新模式。商事制度改革实现“二十三证合一、一照一码”，市场主体达到27.4万户，增长16%。13家省级开发区四项重点改革全部落实。深入推进金融创新，新设立基础设施建设基金、战略性新兴产业引导基金和蓝天基金，县级农村信用联社启动改制5家，建成农商行3家。在全省率先实现全流程电子招投标，率先开展“微改革、微创新”活动，首批推出15项民生改革措施。组建哈院医疗集团，市中医院、市三院、市四院分别牵头组建医联体，试点推进武强、故城医共体改革。作为全省综合配套改革的“试验田”，我市各项改革亮点纷呈，红利加速释放，经济发展的内生动力活力进一步增强。

生态环境明显改善。出台史上最严“1+27”政策体系，实施压煤、优企、抑尘、控车、增绿五大攻坚。积极推进冬季清洁取暖，成功入选北方地区清洁取暖试点城市，完成农村“双代”10万户，桃城区、衡水高新区、滨湖新区实现散煤清零。衡水高新区“西汽东输”工程竣工通汽。在全省率先实现全市域35蒸吨以下燃煤锅炉清零。集中整治“散乱污”企业5018家，4家重污染企业退城搬迁。市区机械化清扫率超过90%。推广新能源车2041标准辆，公交车免费乘坐由上年的4个月延长为5个月。秸秆禁烧工作得到全国人大领导肯定。空气质量达到五年来最好水平，优良天数165天，增加天数和改善率均为全省第一；从去年九月份开始，连续五个月退出全国74个重点城市后十名。加大水生态修复力度，河湖水质普遍提升。确定“一湖十河”名录，全面落实市县乡村四级河长制。南水北调江水切换消纳江水指标量全省第一。衡水湖蓄水量达到近20年最高水平。在全省率先出台“净土行动”工作方案，土壤污染防治工作迈出坚实步伐。全年完成造林34万亩，是省定任务的3.4倍。这一年，环保工作认识空前、力度空前、成效空前，全市上下付出了巨大牺牲、做出了巨大努力，我们终于拨开了头顶上厚重的雾霾，终于让人民群众享受到了更多的蓝天、白云、绿水、阳光。

民生福祉持续提升。强力实施脱贫攻坚突破年活动，921个贫困村实现经济合作组织全覆盖，“京东跑步鸡”等产业扶贫项目受到国家有关部门肯定，3.98万贫困人口脱贫、479个贫困村出列、4个贫困县摘帽。城乡低保标准分别提高12.1%和15.4%。城乡居民医疗保险年人均财政补助标准达到450元。全面改善农村义务教育薄弱学校基本办学条件，工程开工率和竣工率均居全省首位。高中教育继续走在全国前列。农村饮水安全巩固提升工程扎实推进。8所城市公立医院全部实行药品零差率销售，二级医院对口支援乡镇卫生院实现全覆盖。各县市中心城区全部实现集中供暖。成功举办2017衡水湖国际马拉松赛暨全国马拉松锦标赛，第五次获得国内金牌赛事称号。泰华锦业游泳俱乐部创我国省企联办先河，在第十三届全运会中两破亚洲纪录，为体育的职业化改革、带动全民健身趟出了一条新路。平安衡水、法治衡水建设深入实施，刑事治安案件连续五年下降，打击犯罪成效继续稳居全省前列，市信访局被授予“全国信访系统先进集体”荣誉称号。安全生产和食品药品安全总体稳定。国防动员和双拥工作深入开展，军民融合发展持续推进，民兵应急力量建设坚强有力，军转安置工作进一步加强。民生工作全面扎实推进，群众的获得感、幸福感、安全感明显增强。

政府自身建设不断加强。深入推进“两学一做”学习教育常态化制度化，认真抓好机关作风整顿和“一问责八清理”工作。“放管服”改革持续发力。在全省率先成立市级行政审批局，实现市县两级行政审批局全覆盖。在全省率先开展“互联网+稽查”试点。县市区综合检验检测中心实现全覆盖。市县两级城市管理综合执法局全部挂牌，行政执法“三项制度”改革走在全省前列，党政机关法律顾问和公职律师制度全面落实。应急体系建设不断完善。群众服务热线“热度”不减，“信用衡水”上线运行。自觉接受人大法律监督、工作监督和政协民主监督，办理人大代表建议和政协提案564件，办结率100%。干部作风持续转变，忠诚担当、干事创业、奋发作为的氛围更加浓厚。

同时，民族宗教、新闻出版广电、文物保护、人口计生、外事侨务、涉台事务、人民防空、气象、防震减灾、地理信息、邮政通信、援疆、妇女儿童、老龄、残疾人、史志档案等事业也都取得了新进步。

各位代表，过去的一年，在各项工作取得明显成效的同时，我们还完成了几件产生重大影响、具有里程碑意义的大事。一是成功举办河北省首届园林博览会。用短短8个月的时间，打造了全国最大的城市湿地园林、最大的园博花街，举办了国际性的湿地园林与生态城市建设研讨会，开展了招商和系列文艺演出活动，入园游客超100万人次。园博会的成功举办，铸就了“担当、奉献、拼搏、创新”的“衡水园博精神”，成为我市宝贵的精神财富。二是环境高地建设取得新跨越。紧抓雄安新区规划建设机遇，确立了把我市打造成为雄安新区交通“南大门”、产业“新腹地”、生活“近郊区”、环境“后花园”的功能定位。石济客专建成通车，雄商高铁正式启动，我市迈入高铁时代，交通区位优势更加明显，要素集聚能力显著增强。在全省率先推出进一步促进民间投资发展的9条意见和招商引资优惠政策20条，叫响“走遍千山万水、投资首选衡水”的品牌。我市的软硬环境建设步入新高度，在此形势下，我们成功举办衡水北京高校（科研院所）产学研用合作对接恳谈会等16次高规格招商活动，外出招商500多批次，邀请客商来衡对接380批次，累计签约项目139个，总投资2364亿元，是招商引资引智最活跃、最密集的一年，也是成效最明显的一年。三是承办了一系列重要会议和重大活动。召开了第二届夏季央企投资发展论坛等4次国家级会议、全省投资和项目建设观摩调度会等4次省级会议，将我市各领域的成功经验向全省全国宣传推广，衡水对外界的影响力大大增强。上述几件大事的成功举办，让全市上下为之扬眉吐气，发展自信空前增强，外界对衡水的认识有了质的飞跃、质的提升，充分彰显了新时代衡水速度、衡水精神、衡水力量、衡水形象。

各位代表,奋进的历程不同寻常，成绩的取得来之不易。这是省委、省政府和市委正确领导的结果，是全市人民勠力同心、克难奋进的结果，是社会各界和衷共济、通力协作的结果。在此，我代表市人民政府，向全市人民，向人大代表、政协委员，向离退休老同志，向各民主党派、工商联、无党派人士和人民团体，向驻衡部队、武警官兵、政法干警和中省直驻衡各单位，向所有关心支持衡水改革发展的各界朋友，致以崇高的敬意和衷心的感谢!

各位代表，我们也清醒地看到，全市发展中仍然存在不少困难和问题。发展理念还不够超前，眼界还不够开阔，解放思想的任务依然艰巨；经济总量还不够大，质量还不够优，效益还不够高，加快发展和赶超晋位的任务依然艰巨；立市立县的大项目、好项目少，产业层次依然偏低，调结构、转方式的任务依然艰巨；公共服务均等化还有差距，城市管理水平还需提升，民生改善和社会治理的任务依然艰巨；个别部门和工作人员标准不高、作风不实、执行力不强的问题还未根本解决，提升服务水平和优化发展环境的任务依然艰巨。对此，我们一定高度重视，采取有力措施，认真加以解决。

二、2018年经济社会发展的总体要求和主要目标

2018年是贯彻党的十九大精神的开局之年，是改革开放40周年，是决胜全面建成小康社会、实施“十三五”规划承上启下的关键之年，做好今年的工作意义重大。

中国特色社会主义进入新时代，衡水也站在新的历史起点上，正处在一个难得的历史窗口期和战略机遇期。从外部环境看，世界经济继续复苏，我国经济由高速增长阶段转向高质量发展阶段，经济发展总体向好，特别是京津冀协同发展的深入推进和雄安新区的规划建设，为衡水带来了千载难逢的发展机遇。从自身看，我市地区生产总值突破1500亿元，一般公共预算收入突破100亿元，经济总量迈上新台阶，经济社会发展进入了“弯道取直、跨越发展”的快车道；特别是广大干部群众精神面貌积极向上，想发展、谋发展的意识日益强烈，忠诚担当实干的热情日益高涨。我们必须把握机遇、乘势而上，努力推动衡水奋进新时代、引领新发展、开创新局面。

——奋进新时代。就是摆脱落后的新时代，是追求高质量发展的新时代，是全面建成小康社会的新时代，是进行社会主义现代化建设的新时代。我们既要弥补总量不足，解决“盘子小”、发展不充分的问题，也要摆脱产业层次低、生产效益不高、发展质量落后的问题，全力把经济总量做上去，把发展质量提上来。到2020年，我们要与全国全省同步全面建成小康社会；现行标准下农村贫困人口实现脱贫，贫困县全部摘帽。到2035年，基本实现社会主义现代化，全面建成经济强市、美丽衡水，在全省发展大局中的地位和作用进一步提升；其中到2030年“五个衡水”建设取得重大进展，一座生态宜居的美丽湖城呈现在世人面前。到2050年，我国建成富强民主文明和谐美丽的社会主义现代化强国时，全面建成社会主义现代化强市，衡水成为在国内外具有重要影响力的京津冀重要节点城市。

——引领新发展。就是坚决贯彻落实新发展理念，加快建设现代化经济体系，推动质量变革、效率变革、动力变革，走出一条具有衡水特点的高质量发展新路。继续大力实施创新驱动战略，积极推进科技创新与产业发展深度融合，建设京津冀创新发展和产学研合作基地。深化供给侧结构性改革，在推进协调发展中解决好不平衡不充分的问题。进一步扩大生态优势，走绿色发展之路，变绿水青山为金山银山。深化改革开放，不断优化发展软环境，积极引进新技术、新产业、高层次人才，大力提升发展的动力和活力。始终坚持以人民为中心的思想，让改革发展的成果全民共享。

——开创新局面。就是围绕建设京津冀发展环境高地，迈进高质量时代、智慧城市时代、千亿产业时代，打造实力衡水、创新衡水、生态衡水、文化衡水、幸福衡水，大力实施产业转型升级年、改革开放深化年、多城同创提质年、基础设施完善年、生态治理跨越年、扶贫脱贫攻坚年、基层建设夯实年、干部素质提升年“八个年”攻坚活动，努力推动重点领域、重点工作实现突破，引领带动全市经济社会快速健康发展。

2018年政府工作的指导思想是：全面落实党的十九大、十九届一中二中全会、省委九届六次全会和市委四届三次全会精神，坚持以习近平新时代中国特色社会主义思想为统领，按照高质量发展的要求，统筹推进“五位一体”总体布局和协调推进“四个全面”战略布局，坚持创新竞进、协同融合、改革开放、转型升级、提质增效、改善民生、优化环境，全面抓好改革发展稳定各项工作，加快建设新时代经济强市、美丽衡水。

主要预期目标是：地区生产总值增长8%左右，规模以上工业增加值增长6%左右，固定资产投资增长8%左右，一般公共预算收入增长8%以上，全部财政收入跨上200亿元台阶，出口总值、实际利用外资分别增长5%，社会消费品零售总额增长10%左右；城镇、农村居民人均可支配收入分别增长9.5%左右和10.5%左右；城镇登记失业率控制在4.5%以内；居民消费价格指数涨幅控制在3%左右；万元生产总值能耗、PM2.5平均浓度降幅及化学需氧量、二氧化硫、氨氮、氮氧化物减排完成省下达的目标任务。

做好今年工作，必须牢牢把握发展这个第一要务，坚持项目立市不动摇，以持续不断的有效投资提升衡水跨越赶超的“加速度”；必须牢牢把握高质量发展这个根本要求，坚持以供给侧结构性改革为主线，用创新催生衡水跨越赶超的“新动能”；必须牢牢把握疏解北京非首都功能这个“牛鼻子”，在京津冀协同发展中打造衡水跨越赶超的“新引擎”；必须牢牢把握人与自然和谐共生这一发展要求，坚持生态严管严治，守住衡水跨越赶超的“生命线”；必须牢牢把握以人民为中心的发展思想，把满足人民美好生活需要作为民生重点，在共建共享中提高衡水跨越赶超的“普惠度”。

三、2018年重点工作任务

实现今年目标任务，要抓好九个方面重点工作：

（一）深化供给侧结构性改革，着力推进产业转型升级。围绕做大做强“4+4”主导产业，大力实施产业转型升级年，在“破”“立”“降”上下功夫，坚持调强存量与调优增量并举，努力在新旧动能转换上攻坚突破。

大力破除无效供给。巩固“散乱污”企业整治成果，抓好僵尸企业出清、市区“退二进三”工作，严把项目关口，坚决不上落后产能和低质项目。

加快科技创新步伐。全面启动战略性新兴产业三年行动计划，围绕发展信息产业、高端装备制造、新材料、新能源、节能环保和生物医药等新兴产业，加快引进高新技术和高科技项目。持续跟进与中科院、北京大学、清华大学、中国农科院等战略合作协议落实，用好京南国家科技成果转移转化示范区政策机遇，积极推进产学研深度融合。打造“中科院+”“院士+”“千人计划+”协同创新共同体，推动衡水科技谷建设，办好国家“千人计划”专家衡水湖论坛，年内新建产业技术研究院、工程技术研究中心、院士工作站等创新服务平台8家以上，新增省级以上众创空间、星创天地、科技企业孵化器5家以上。实施高新技术企业和科技型中小企业三年双倍增计划，力争新增高新技术企业45家，新培育科技型中小企业750家。用好省、市“科技创新券”，支持科技型小微企业发展。市县两级财政科技投入和全社会研发投入占比均要达到全省平均水平。实施企业专利提升计划，年内新增100家专利企业，新增发明专利150件以上。

推动企业提质增效。加快技改升级步伐，推动传统产业向高端化、链条化、智能化、绿色化发展，全年力争实施省市重点技改项目不低于100项。认真落实支持工业设计发展的政策措施，高标准谋划市级工业设计中心，每个特色产业集群都要对接一流设计公司。扎实推进质量兴企，按照标准、设计、质量、品牌、信誉“五位一体”工作格局，以提升产品质量为重点，实施百项新产品开发、百项名牌产品培育工程，力争申报省中小企业名牌产品15项以上。深入推进企业经营者素质提升和现代企业制度建设两大工程，积极开展企业“双对标”行动，在全国范围内确定行业标杆，在市域内确定企业标杆，持续提升管理水平。加大企业上市奖励力度，力争新上市挂牌企业10家以上。落实工业企业保运行的8条措施，强化督导调度和企业帮扶，确保工业经济平稳运行。

大力降低实体经济成本。开展“降费减负”专项行动，在落实省“降税、减证、提标”三项措施的基础上，研究出台一批易操作、能解渴的政策措施。举办农业产业化、外贸、科技、旅游等专题银企对接活动，实施重点企业“金融港”护航计划，引导企业通过发行公司债、企业债、私募债、中期票据等募集资金，提高直接融资比重，降低融资成本。进一步降低制度交易、用能、用工、物流等成本，打造企业成本洼地、投资环境高地。

壮大民营经济。毫不动摇地鼓励和支持民营经济发展，坚持“非禁即准、平等待遇”，坚决取消针对民间投资的歧视性附加条件和隐性条款。全面落实促进民营经济发展的各项优惠政策，拓宽融资渠道，做好用地保障，建强服务平台，推动新一轮民营经济大发展、快发展。企业家是社会财富的创造者、创新活动的实践者，要尊重、支持和爱护企业家，激发和保护企业家精神，使广大企业家感受到亲商、安商、富商的衡水态度、衡水温度。

（二）积极推进京津冀协同发展，着力抓好项目建设。围绕打造“一枢纽、四基地”目标，以项目建设为主抓手，加快在对接、服务、融入中发展自己。

推动产业对接。紧盯京津食品加工、纺织服装、装备制造、新型建材等行业动向，开展精准对接。推进30项京津冀协同发展重点项目建设，抓好与中冶、中核工、中建、中铁等30个央企重点合作工程。支持枣强中关村产业协同创新基地、冀州旅游设施装备产业园、阜城中华老字号食品产业园、深州京津冀产业协作园、故城雄安乳胶产业园等共建园区。依托滨湖新区，积极谋划建设北京非首都功能疏解集中承载地。依托基础教育资源优势，吸引京津高校和科研院所向我市转移，打造产城教融合发展试验区。全方位推进与京津医疗机构的合作，促进京津优质医疗资源向我市延伸拓展，以大健康新医疗为驱动，着力建设“滨湖健康休闲养生产业园”。积极对接雄安新区，加强合作，借势发展。

促进交通一体化。瞄准打造京南综合交通枢纽，编制综合交通枢纽规划。加快国省干线升级改造步伐。加快石衡高速、邯港高速建设，做好雄商高铁、石衡沧港城际铁路前期工作，争取早日开工。谋划衡水联接雄安新区的快速通道。深入推进京津冀城乡客运一体化。

狠抓重点项目。持续掀起大上项目、上大项目的新热潮。大力实施“3233”工程，突出抓好300项左右省市重点项目，新开工亿元以上项目200项以上，重点推进30项重大产业项目和30项市政重点工程。坚持“三个机制”，突出“三个支持”，搞好“三个服务”，开展集中开工、专项督查、观摩拉练等系列活动，落实层级分包调度、主要领导亲自协调、要素服务保障等制度，着力解决项目谋划浅、前期长、推进慢等问题，打通项目快速落地、顺利实施的绿色通道。正大肉鸡全产业链等30个项目年内确保投产；和平铝业铝型材深加工等70个项目争取尽早开工；中粮集团30万吨果糖和30万吨乙醇等20个项目加快完善前期要件，力争取得实质性进展。年内新谋划立市立县重大项目100项，争取更多的县市区能有百亿元项目。

（三）聚焦拓展升级，着力加快现代服务业发展。推动生产性服务业向专业化和价值链高端延伸，生活性服务业向精细化和高品质升级，壮大规模、提高档次，加速形成新的经济增长点。

转型提升传统商贸业。推动消费升级，积极引进新的商业业态，促进国内知名品牌落地建设。实施“互联网+衡水名优新特产”行动，支持京东“中国特产衡水馆”运营，推动传统零售企业线上线下互动。实施电子商务示范引领工程，培育一批商业模式成熟的电商龙头企业。依托农村电商网点，推进“万村千乡”农家店信息化改造和“农超对接”电商应用。支持“吉鲜蜂”“淘实惠”等本地电商平台，推进电商进社区。

打造“文旅农”融合的特色旅游业。坚持全域旅游发展理念，办好市第二届旅发大会，持续打造“五大旅游片区”。丰富旅游产品，加强宣传营销，打造“衡水旅游小镇”新景区，大力发展环湖康养健身游、儒乡研学游、乡村休闲游、文化创意游，打造京南“醉美湿地、大儒之乡”休闲旅游目的地。

壮大做强文化产业。突出董仲舒文化研究，推进环衡水湖新兴文化产业核心区、中北部文化装备制造业集聚区、董子故里文化集聚区、大运河历史传统文化产业带和滏阳河文化带“三区两带”建设，推进十大特色文化产业发展，建设特色文化小镇。推进政府购买社会公共文化服务，进一步丰富文化供给。

积极发展新兴服务业。围绕建设全国现代商贸物流基地，加快推进新发地饶阳农副产品物流园等重点项目，加快融入环首都一小时鲜活农产品物流圈。抓好安平丝网、大营皮毛等特色产业公共服务平台建设，培育研发设计服务产业。抢抓石济客专开通机遇，加快推动高铁商务区建设，着力发展服务外包、总部经济等新兴业态。大力发展金融服务业，优化金融生态环境，着力引进基金管理公司等非银行金融机构；加快推进县级农村信用联社改革，支持衡水银行、衡水农商行等金融机构做大做强，推动8家村镇银行尽快落地开业。推进“互联网+”行动计划，打造以“大智移云”为核心的信息服务业。大力发展健康养生、养老服务、社区服务等生活性服务业。

（四）实施乡村振兴战略，着力推进农业农村现代化。坚持规划先行、有序推进，落实好“产业兴旺、生态宜居、乡风文明、治理有效、生活富裕”总要求，努力走出一条有衡水特色的乡村振兴之路，使我市农业农村工作继续走在全省前列。

大力发展高质量绿色农业。围绕在全省率先建设绿色农业示范市，着力实施七大工程。实施农业结构调整优化工程，积极构建粮经饲三元结构，大力发展节水农业，开展季节性休耕，建设畜牧、蔬菜、果品三大特色产业优势区。实施农产品质量安全保障工程，推进农业标准化生产，开展化肥农药使用量零增长行动，加强农业投入品监管和疫病防控，健全农产品质量追溯体系，推进农产品“三品一标”认证，绿色食品认证企业达到8家。实施现代农业园区提档升级工程，支持安平生猪产业园创建国家现代农业产业园，支持饶阳创建国家农业科技园区。实施农业科技支撑工程，建设农业科技成果转化基地，推进“互联网+现代农业”行动计划。实施农业品牌创建工程，培育区域公用品牌2个，省级农业名牌3个。实施龙头带动工程，推进农产品加工项目建设，支持龙头企业建设中央厨房。实施秸秆和畜禽养殖废弃物资源化利用工程，利用2-3年时间，在全省率先实现市域全覆盖。

加快推动农民全面发展。推动农户与现代农业有机衔接，积极培育家庭农场、农民合作社等新型农业经营主体，力争全市注册家庭农场达到7600家，规范化运行的农民合作社达到1600家，股份合作制经济组织达到4700家，带动农户22万户。推广阜城“八统一分”经营模式，搞好产前、产中、产后全过程服务。加强农村基层基础工作，抓好新型职业农民培育，推动农村移风易俗，培育文明乡风、良好家风、淳朴民风。

建设乡村振兴示范点。推进“三区同建”，以县城、乡镇政府驻地和产业园区周边为重点，结合产业园区、生态园区建设，扎实推进新型社区建设，全市年内新谋划启动示范点30个以上。实施农村人居环境整治三年行动，推进村庄绿化、厕所革命、农村垃圾污水治理，争创一批农村人居环境整治示范县，打造一批美丽宜居乡村。制定并大力实施“四好农村路”建设计划，把农村公路建成农民的“暖心路”。

（五）注重城乡统筹，着力推进新型城镇化。抢抓建设京津冀世界级城市群和省“支持衡水统筹城乡发展，建设生态宜居的滨湖园林城市”等机遇，加快新型城镇化进程。

做大做强做美中心城市。坚持走内涵式城市发展道路，围绕“四个一百”目标，按照“一湖三区”组团式空间定位，统筹老城区和新城建设，进一步提升中心城市的品位和承载力。启动总体规划修编，完成高铁区域和南部新区城市设计。突出湖城特色，科学规划城市绿地系统，加快滏阳一路、西外环路等道路绿化、亮化、美化。加快海绵城市、绿色节能城市、智慧城市建设，建成启用衡水市大数据中心。开展基础设施完善年，全力推进市政基础设施重点工程，实施市区大庆路东延、人民路东延、振华路南延、植物园道路贯通、南部新区综合管廊及路网等道路工程；加快推进南华再生水厂建设；改造报社街等菜市场，市区新建公厕32个；新建生活垃圾处理厂投入使用。野生动物园、植物园、孔颖达文化公园、宝云公园、滏东公园建成并对外开放，改造提升园博园、人民公园、滨湖公园、盐河故道公园，满足市民对休闲活动场地的需求。继续办好湿地园林与生态城市建设研讨会。

推动县城和小城镇建设。持续开展县城建设攻坚，抓好各县城的城市双修工作，加强公共场馆和全民健身中心建设，加大星级公园创建力度，全面提升县城基础设施和公共服务功能。今年，力争县城四星级公园全覆盖，全省县城建设后30名的县出列。加快小城镇建设，完善镇区道路、供排水、垃圾处理、文化教育、卫生体育等各项设施，提高人口吸纳能力，全市重点培育9个特色小城镇，每个县市区重点抓好1-2个中心集镇。

开展“多城同创”活动。着眼城市高质量发展，大力实施多城同创提质年，统筹创建全国质量强市示范城市、国家园林城市、国家森林城市、国家节水型城市、省级文明城市、省级卫生城市、全省优先发展公共交通示范城市、省级食品安全示范城市。通过精准对标、靶向施策、市县联动、全域创建，推动城市承载能力、治理水平大幅提升，城乡风貌、文明程度明显改观，使我们衡水成为京津冀城市群中风景秀丽、宜居宜业的璀璨明珠。

（六）立足激发动力活力，着力深化改革开放。实施改革开放深化年，充分发挥全省综合配套改革试验市的作用，加大改革开放的广度和深度，进一步释放红利、激发活力、提升竞争力，争当全省改革开放的排头兵。

深化重点领域改革。突出抓好“放管服”改革，衔接落实好国务院、省政府清理规范的行政审批事项，推动“双随机一公开”监管模式全覆盖，推行“互联网+政务服务”；推进市县两级行政审批局、市县乡三级综合行政执法机构规范化标准化建设，加快市县公共资源交易中心全过程电子化，完成市民公共服务中心搬迁，完善中介服务管理中心运行机制；深化“多证合一”改革，加快“一照通”审批监管协同平台建设。衔接落实好第二轮土地承包到期后再延长30年的政策，完成颁证扫尾工作；全面开展农村集体资产清产核资工作，有序推进集体经营性资产产权制度改革，推广饶阳做法，多措并举壮大集体经济，力争用1-2年时间“消灭”空白村。落实《支持衡水高新区加快发展的十条措施》，衡水高新区今年力争晋升国家级高新区。统筹抓好经营性事业单位转企、经济发达镇行政管理体制改革扩面、组建衡水中学教育集团、妥善处置房地产领域遗留问题、深化医药卫生体制改革、安全生产领域改革等重点改革攻坚任务。

精准开展招商引资。大力开展“迎商”活动，瞄准高端高质项目精准招商，着重从京津引进产业转移和科技成果转化项目，从珠三角引进服务业和新兴产业项目，从长三角引进制造业补链强链及高端制造项目。精心组织京津、长三角、珠三角等区域招商会，积极参加上级组织的重大招商活动，办好衡水湖国际商务洽谈会等自办招商活动，争取做到季季有主题、月月有活动，务求招商实效。

努力扩大对外开放。实施“外贸精准帮扶攻坚行动”，切实帮助企业解决融资退税、通关报检、市场开拓等难题。推进丝网、裘皮国家级外贸转型专业型示范基地和精细化工省级出口基地建设。积极对接“一带一路”战略，全方位拓展对外合作交流，推动公铁交通装备、物流输送设备等产业“走出去”。

（七）改善生态环境，着力建设更高水平的美丽衡水。围绕打造京津生态屏障保护基地，实施生态治理跨越年，坚定走绿色发展、生态优先之路，推动生态优势向高质量发展优势转化。

坚决打赢蓝天保卫战。坚持市县同步、城乡一体、全民共治，制定大气污染治理3年作战计划和“退后十”攻坚行动方案。强力推进清洁取暖、电厂改造、VOCs治理、工业达标、清洁降尘、控车减油、面源治理、科技治霾“八个专项行动”。积极探索农村多元化清洁能源替代之路，年内新增15万户以上，具备地热资源的乡镇启动地热能集中供暖。完成衡丰、恒兴电厂湿烟羽治理工程，35蒸吨以上燃煤锅炉全部达到排放限值标准。开展工业企业提标升级改造，加快污染企业退城搬迁。主城区保洁面积扩大一倍，深化建筑施工扬尘专项整治。继续实行取暖季公交免费乘车，加快构建城市风道系统。今年我市的空气质量排名，要力争率先稳定退出全国74个重点城市后十名。

统筹推进水污染和土壤污染治理。全面落实“水十条”，巩固“河长制”，推行“湖长制”，实施河湖生态修复、工业废水全面达标、生活污水治理、规模化畜禽养殖废水治理和饮用水水源保护五大工程，全面提升水环境质量。建立衡水湖统一管理机制，完成保护区违法违规建设活动整改，推进衡水湖整体生态治理修复，完成国际重要湿地申报工作。实施主城区水系生态修复工程，完成黑臭水体治理。巩固拓展地下水超采综合治理成果，2020年基本实现地下水采补平衡。加快城乡供水一体化。全面落实“土十条”，启动土壤环境数据库建设，开展耕地土壤环境质量差别划分。严格保护耕地，改进占补平衡管理方式，加强土地执法检查。

加快生态文明制度体系建设。积极探索以绿色发展为主导的考核体系，试行开展生态损害赔偿制度，建立“三线一单”硬约束。推进政府和社会资本合作、环境污染第三方治理模式，健全环保信用评价制度，推行企业自行监测数据公开，建立违法排污黑名单制度。充实和加强基层环保力量。广泛开展创建节约型机关、绿色家庭、绿色学校、绿色社区和绿色出行等行动，形成崇尚生态文明的良好社会风尚。

大力推进造林绿化。围绕构建京津冀生态环境支撑区，编制造林绿化规划，重点发展优质果品、林板一体化、生态文化旅游和休闲康养、特种经济林、林下经济、花卉苗木六大产业集群。深入推进“三环一覆盖一运河”造林绿化工程，打造环衡水市区、环县城、环乡镇村庄综合防护林带，实现城市城镇建成区、乡镇村庄所在地裸露土地绿化美化香化全覆盖，推进大运河生态景观长廊建设。全年造林20万亩，森林覆盖率达到32.6%。

（八）坚持以人民为中心，着力发展民生和社会事业。人民对美好生活的向往，是我们始终不渝的奋斗目标。我们将持续加大民生投入，针对人民群众关心的问题精准施策，尽力而为、量力而行，让人民群众得到更多看得见、摸得着的实惠。今年计划重点实施12项民生实事，待这次大会票决通过后，将逐个制定专项实施方案，确保高标准、高质量完成。

大力实施扶贫脱贫攻坚年。坚决扛起脱贫攻坚重大政治责任，深入开展脱贫攻坚作风建设年活动。准确把握扶贫脱贫新变化新要求，实施“三落实三提高”工程，持续改善贫困村生产生活条件。对贫困人口因户施策，精准确定脱贫路径，对有劳动能力的强化产业就业扶持，以稳定增收促进稳定脱贫；没有劳动能力的采用兜底式扶贫。全年力争实现1.78万贫困人口脱贫、80个贫困村脱贫出列、武强和故城两县脱贫摘帽。消除贫困既是我们的庄严承诺，更是我们的历史使命，离目标越近，越要保持定力、加速冲刺，通过全市上下的共同努力、不懈奋斗，彻底结束我市有贫困县的历史，取得脱贫攻坚战的决定性胜利。

优先发展教育事业。着力解决幼儿园总量不足、师资力量不够的问题，盘活主城区学前教育资源，对住宅区已配套建设的幼儿园全部由政府统筹管理，更多地用于举办公立幼儿园或普惠性民办幼儿园。新建冀州第二幼儿园。努力缓解市区中小学大班额问题，新建裕华路小学、新华小学、郑里马小学，改扩建魏家屯镇学校。做大做强高中教育，规范办学行为，提升办学品质。加大教师培训力度，促进民办教育规范健康发展。谋划衡水学院新校区建设，推动衡水学院、衡水职业技术学院实现特色优质发展。

提高就业质量。实施高校毕业生就业创业促进计划和基层成长计划，扎实做好重点人群就业服务，支持农村劳动力转移就业和农民工返乡创业，严厉打击拖欠农民工工资行为，组织供需见面会20场以上，城镇新增就业4.2万人。

加快多层次社会保障体系建设。深入实施全民参保计划，推进机关事业单位养老保险制度改革。完善最低生活保障和社会救助制度，健全农村留守儿童、妇女、老人关爱服务体系，加大对残疾人补贴力度，继续提高城乡居民最低生活保障标准。

保障群众基本住房需求。抢抓国家政策窗口期，实施新的三年棚户区改造攻坚计划，年内完成1.2万套。以低保、低收入住房困难家庭为重点，加大公租房保障力度。加快城中村改造和老旧小区改善。年内完成农村危房改造1200户。

提高医疗保障服务水平。加强分级诊疗、现代医院管理、全民医保、药品供应保障、综合监管五项制度建设。市妇幼保健院新院区、市第二中医院病房楼年内投入使用，哈院南院区、衡水高新区医院开工建设。市中医院新院区筹建工作取得实质性进展。市四院力争晋升三甲医院。

加快文化体育事业发展。倡导全民阅读，衡水大剧院、市图书馆向群众开放，市博物馆加快布展。支持民营博物馆发展。组织形式多样的文化惠民演出活动。大力开展全民健身活动。高标准举办2018衡水湖国际马拉松赛暨全国马拉松锦标赛，争创国际田联银标赛事。谋划建设体育主题公园。积极争取河北女子篮球队落户衡水，提高我市竞技体育的发展水平，带动群众性体育健身活动的开展。侯卫东官场笔记

（九）打好防范化解重大风险攻坚战，着力提升社会治理水平。有效防控金融风险，坚决守住不发生系统性、区域性金融风险的底线。有效防控安全生产风险，深入开展安全生产隐患大排查大整治大执法攻坚行动，促进安全生产形势稳定好转。强化食品药品全过程监管，确保人民群众舌尖上的安全。有效防控社会风险，集中开展社会矛盾排查化解专项行动，最大限度将矛盾纠纷化解在基层。加快社会治安防控体系建设，强力开展扫黑除恶专项斗争，继续推进天网工程，严厉打击涉众型经济犯罪、电信诈骗等违法犯罪行为。加强社会诚信体系建设。创新现代乡村治理体制，完善社区治理体系。落实改革强军战略部署，加强国防动员和后备力量建设，推进军民融合深度发展，增强全民国防意识，扎实做好民兵工作，加强退役军人管理服务保障工作，不断巩固发展坚如磐石的军政军民关系。充分发挥工会、共青团、妇联等人民团体桥梁纽带作用。继续做好民族宗教、新闻出版广电、人口计生、外事侨务、涉台事务、人民防空、气象、防震减灾、地理信息、邮政通信、援疆、妇女儿童、老龄、残疾人、史志档案等工作。

四、加强政府自身建设

新时代、新目标、新任务，对政府自身建设提出了新的更高要求。发展高质量，必须依靠工作高标准来实现。我们必须以习近平新时代中国特色社会主义思想为指导，深入学习贯彻党的十九大精神，不忘初心再出发，勇挑重担立潮头，努力开创政府各项工作新局面。

讲政治顾大局，坚决维护核心。切实增强“四个意识”，自觉同以习近平同志为核心的党中央保持高度一致，坚决维护党中央权威和集中统一领导，坚决贯彻中央、省和市委各项决策部署，确保令行禁止、政令畅通。推动“两学一做”学习教育常态化制度化，扎实开展“不忘初心、牢记使命”主题教育，坚定不移用习近平新时代中国特色社会主义思想武装头脑、指导实践、推动工作。

抓工作高标准，打造学习政府。提升工作目标，调优工作状态，激发工作热情，敢为人先，唯旗是夺，把全省领先作为工作追求，在全国范围内找标杆、学榜样。按照建设高素质专业化干部队伍的要求，弘扬专业精神，加强能力建设，增强施政本领，在全市政府系统营造善于学习、勇于实践的浓厚氛围。大兴调查研究之风，坚持学以致用，在学中干、干中学，不断增强工作的预见性、针对性和实效性，牢牢把握工作的主动权。

履职守敢担当，打造责任政府。把造福人民作为最大的政绩，把心思用在干事创业上，把工作做到老百姓的心坎上。困难面前敢于开拓，矛盾面前敢抓敢管，风险面前敢担责任。对确定的任务目标，坚决推进到位；向人民作出的承诺，坚决如期兑现，不搞遥控指挥、不做甩手掌柜、不搞“击鼓传花”，做到敢干事、真干事、会干事、干成事，真正担负起兴一方经济、富一方百姓、建一方文明、保一方稳定的政治责任。

强作风重落实，打造效能政府。加强效能建设，优化政务环境，以更彻底的放权、更严格的监管、更精准的服务，推动职能转变，释放市场活力，让企业和群众少跑腿、好办事。不驰于空想、不鹜于虚声，一步一个脚印，踏踏实实地干好工作。坚持工作项目化、一线工作法，加大督导力度，健全问责机制。以盯住干、马上办、抓到位的劲头推进工作，始终保持旺盛的工作热情，撸起袖子加油干，苦干实干拼命干，用我们的不懈努力提升全市人民的幸福指数。

遵法纪严要求，打造法治、廉洁政府。把政府的一切工作纳入到法律的框架之下，推动形成办事依法、遇事找法、解决问题用法、化解矛盾靠法的良好法治环境。深化政府法律顾问、公职律师制度，实现重大事项合法性审查全覆盖。自觉接受人大的法律监督、工作监督和政协的民主监督,广泛听取民主党派、工商联、无党派人士、人民团体意见，认真办理人大代表建议和政协提案。加强廉政建设和反腐败斗争，严格落实中央八项规定和廉洁自律各项规定，加大审计监督力度，驰而不息反对“四风”，让人民群众从政府各项工作中感受到清风正气。

各位代表！新时代催人奋进，新征程任重道远，让我们更加紧密地团结在以习近平同志为核心的党中央周围，在省委、省政府和市委的坚强领导下，不忘初心，牢记使命，忠诚履职，砥砺前行，为建设新时代经济强市、美丽衡水而努力奋斗！
